# Optical dispersion control in surfactant-free DNA thin films by vitamin B_2_ doping

**DOI:** 10.1038/s41598-018-27166-x

**Published:** 2018-06-19

**Authors:** Bjorn Paulson, Inchul Shin, Hayoung Jeong, Byungjoo Kong, Reza Khazaeinezhad, Sreekantha Reddy Dugasani, Woohyun Jung, Boram Joo, Hoi-Youn Lee, Sungha Park, Kyunghwan Oh

**Affiliations:** 1 Photonic Device Physics Laboratory, Institute of Physics and Applied Physics, Yonsei University, Seoul, 120–749 South Korea; 2 Samsung Electronics, Hwasong, Gyeonggi-do, 18448 South Korea; 3 Harvard Medical School, Boston, Massachusetts, 02115 USA; 4 Wellman Center for Photomedicine, Massachusetts General Hospital, Boston, Massachusetts, 02114 USA; 5 Sungkyunkwan Advanced Institute of Nanotechnology (SAINT) and Department of Physics, Sungkyunkwan University, Suwon, 440-746 South Korea; 6 Space Optics Research Center, Korea Research Institute of Standards and Science, Daejeon, 34113 South Korea

## Abstract

A new route to systematically control the optical dispersion properties of surfactant-free deoxyribonucleic acid (DNA) thin solid films was developed by doping them with vitamin B_2_, also known as riboflavin. Surfactant-free DNA solid films of high optical quality were successfully deposited on various types of substrates by spin coating of aqueous solutions without additional chemical processes, with thicknesses ranging from 18 to 100 nm. Optical properties of the DNA films were investigated by measuring UV-visible-NIR transmission, and their refractive indices were measured using variable-angle spectroscopic ellipsometry. By doping DNA solid films with riboflavin, the refractive index was consistently increased with an index difference Δn ≥ 0.015 in the spectral range from 500 to 900 nm, which is sufficiently large to make an all-DNA optical waveguide. Detailed correlation between the optical dispersion and riboflavin concentration was experimentally investigated and thermo-optic coefficients of the DNA-riboflavin thin solid films were also experimentally measured in the temperature range from 20 to 85 °C, opening the potential to new bio-thermal sensing applications.

## Introduction

Bio-compatible photonic materials are being intensively investigated for their high potential in biomedical detection, monitoring, and therapeutic applications^1,2^. Among these materials, deoxyribose nucleic acid (DNA) thin solid films have recently been found to have unique optoelectronic applications, including organic light emitting diodes, solar cells, optical amplifiers, optical modulators, and nonlinear saturable absorbers^3–7^. As a host material, DNA also provides a stable structure which is highly amenable to the binding of small organic molecules by one of three processes: intercalation, groove binding, and ionic bonding^8–10^ to allow functionalizing dopants embedded in DNA solids^8^. In order to overcome the high surface tension of water-based DNA solutions in conventional spin-coating, most previous reports have focused on complexes consisting of DNA and a surfactant, most commonly cetyltrimethylammonium (CTMA)^11–14^. However, incorporation of cationic surfactants could result in unwanted and sometimes uncontrollable ion-exchange interactions with ionic dopants^15^, which might limit further doping of DNA with functional materials. The inherent toxicity of these surfactants may also raise issues related with long-term biomedical compatibility and safety in practical applications.

In order to avoid these difficulties caused by potentially hazardous surfactants, several research groups have deposited surfactant-free DNA thin solid films and devices by evaporating aqueous DNA solutions. Recently Lee *et al*. have demonstrated biodegradable DNA micro-needles for drug delivery cast from salmon DNA^16^, while Rikimaru *et al*. have proposed a bilayer DNA-chitosan surgical tape^17^. In optics, Śmiałek *et al*. recently measured the density of surfactant-free DNA films using UV-visible interferometry, and those measurements were used to estimate the optical and electrical properties of DNA films^18^, while Samoc *et al*. measured the refractive index of dried and spun thin films of DNA by the prism coupling method, describing the anisotropic index dispersion using a Sellmeier-type formula at two wavelengths, λ = 632.8 and 814 nm^19–21^. More recently, Nizioł *et al*. have used an ellipsometric method in the ultraviolet to characterize the hydration behaviour and thermal breakdown of DNA thin films under heat treatments^22^. Despite prior efforts, research to control the refractive index of DNA film in a consistent and systematic manner has been very scarce.

Control of the optical dispersion in thin solid films is a fundamental prerequisite to optical waveguide research, in which the core should have a slightly higher refractive index than the cladding to guide light. In fact, most silica optical waveguides are composed of Ge-doped silica core and silica cladding, where the index difference Δ*n* is provided by Ge-doping^23^. Therefore, in biocompatible all-DNA optical waveguides it is highly desirable to control the refractive index by adding miscible biocompatible dopants into surfactant-free DNA films to form the core and the cladding.

In this study, we experimentally demonstrated a new route toward systematic, consistent, and repeatable control of the refractive index of a surfactant-free DNA thin solid film by doping DNA with riboflavin, also known as vitamin B_2_, for the first time to the best knowledge of the authors. This unique method may provide new potential all-DNA photonic devices that are inherently bio-compatible to be used in various applications embeddable in living tissues. In addition, riboflavin has recently shown an optical gain^24,25^ in gelatins, so that all-DNA active solid state devices could be further pursued by utilizing this new method.

**Figure 1:**
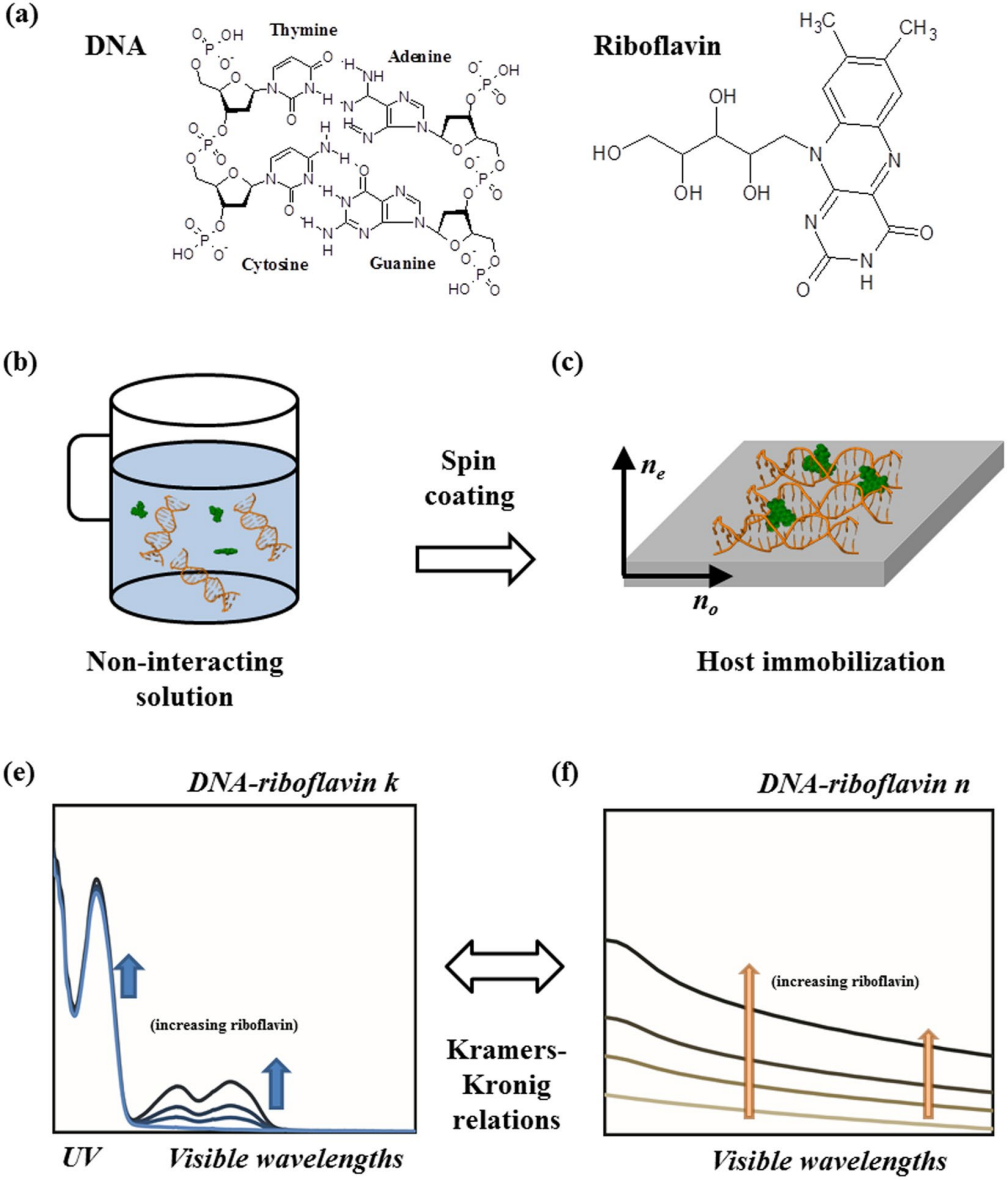
Schematic diagram of controlling the refractive index of surfactant-free DNA thin solid film by doping riboflavin. (**a**) Molecular structures of DNA and riboflavin. (**b**) a mixture of DNA (orange helices) and riboflavin (green dots) in a aqueous solution, (**c**) a deposited thin solid DNA film, where riboflavin fills out the DNA links, stabilizing the polymer structure, (**e,f**) Increasing oscillator strengths by doping riboflavin results in increases in the refractive index, following the Kramers-Kronig relations.

A schematic of the principle for control of the refractive index of surfactant-free DNA thin solid film is shown in Fig. 1. DNA and riboflavin molecular structures are shown in Fig. 1(a). Since DNA and riboflavin are at a relatively low concentration in aqueous solution, they were not strongly interacting, as shown in Fig. 1(b). However, when the solution is spin-coated and dried on a substrate to make a surfactant-free DNA thin solid film, as in Fig. 1(c), riboflavin molecules find appropriate binding sites within DNA and get immobilized in the film, which results in a strong modification of optical properties of DNA films. In addition to the characteristic UV absorption of DNA near λ = 260 nm (refs.^10,21^), riboflavin adds absorption bands in the visible spectral regions^25,26^ as shown in Fig. 1(e). By the Kramers-Kronig relation, changes in the optical absorption of a material directly imply changes in the refractive index at energies below that of the absorption peak^27^. This is the key to systematic control of the refractive index of surfactant DNA film proposed in this study, as shown in Fig. 1(f).

In this paper, we thoroughly investigated the impact of riboflavin doping on the refractive index dispersion in fully bio-compatible surfactant-free DNA thin solid film, for the first time. Detailed thin film fabrication processes, optical measurement results, and their implications are explained in the following sections.

## Experimental Results

### DNA and riboflavin in aqueous solutions

We used commercially available salmon DNA sodium salt (Ogata Research Laboratories Ltd., Japan) and riboflavin (Sigma-Aldrich) with a high purity. DNA and riboflavin were dissolved separately in deionized water. Aqueous solutions of riboflavin were mixed with DNA aqueous solutions resulting in solutions of 0.35 wt. % DNA and 10~130 μM riboflavin in deionized water. Prior to thin film deposition, refractive indices, absorption, and fluorescence spectra of DNA and DNA-riboflavin aqueous solutions were characterized to investigate interactions between DNA and riboflavin in the solution state.

**Figure 2:**
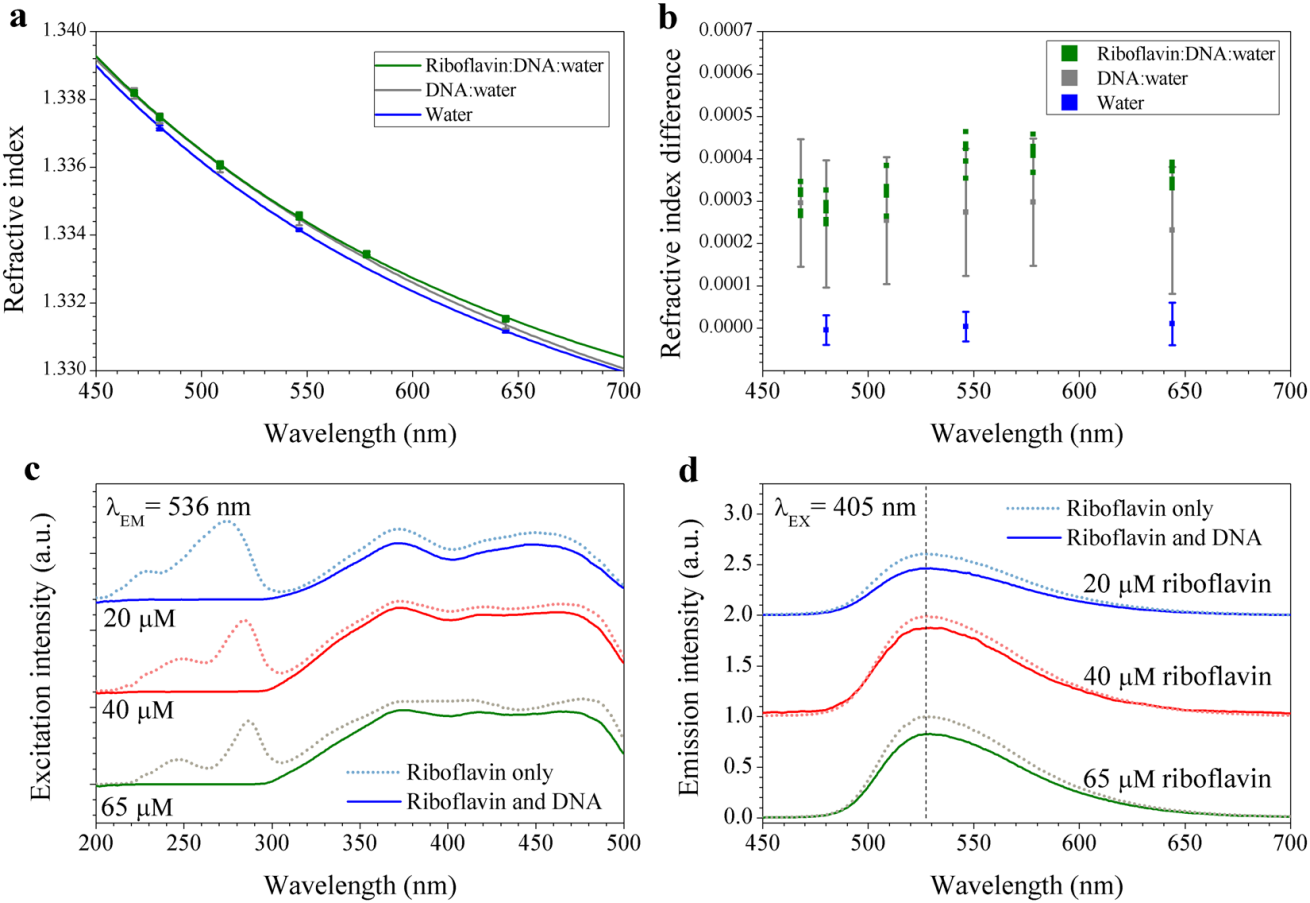
Optical properties of DNA and DNA-riboflavin in aqueous solutions. (**a**) Refractive index dispersion in the visible to near IR spectral range. (**b**) Refractive index increments of DNA and DNA-riboflavin solutions relative to that of de-ionized water. The concentrations of DNA and riboflavin were 0.25 wt. % and 10–130 μM, respectively. (**c**) Fluorescence excitation spectra and (**d**) emission spectra of riboflavin aqueous solutions are compared with and without DNA. Error bars in (**a**) and (**b**) are standard errors in the mean (SEM) in the measured angle.

The prism minimum deviation angle method^28^ was used to determine the refractive indices of aqueous DNA and riboflavin-DNA solutions in the visible range, and the results are shown in Fig. 2(a,b). In the visible range, the refractive index dispersion was dominated by the dispersion of deionized water, and the measurements showed a statistical error of ±0.0001 due to thermal expansion. Within this experimental error, no significant contribution of riboflavin to the refractive index dispersion of DNA aqueous solutions was observed. Fluorescence spectrophotometry measurements were used to detect changes in the fluorescence of the riboflavin molecule caused by the interaction with DNA in solution, and the results are shown in Fig. 2(c and d). The riboflavin molecule has been known to show broad excitation peaks in the near ultraviolet, which result in a typically green fluorescence around 530~550 nm^29^. The fluorescence intensity decreased with DNA concentration, which is mainly due to the optical absorption of DNA from 200 to 300 nm, and scattering in the DNA solution. Shifts in excitation band spectral positions in the liquid samples were within experimental errors. Excitation spectra of riboflavin did not change significantly with addition of DNA at low concentrations, and the emission spectra of riboflavin in DNA solution showed neither significant spectral shift nor meaningful changes in full width at half maximum (FWHM). These observations demonstrate that DNA and riboflavin were not strongly interacting in aqueous solution due to their relatively low concentrations, as schematically shown in the left of Fig. 1(b), although the possibility of long-range interactions between DNA and riboflavin remains.

### Optical dispersion in surfactant-free DNA thin solid films

**Figure 3:**
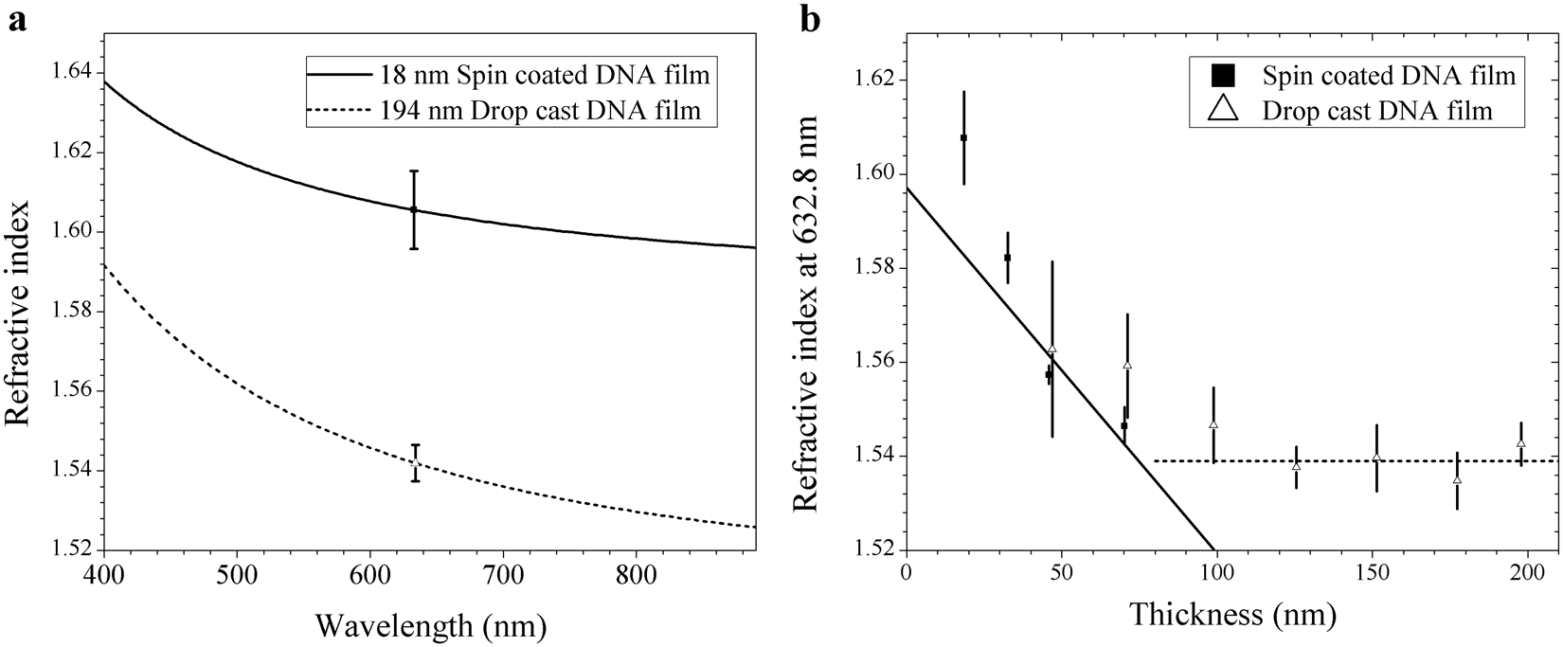
(**a**) Comparison of the refractive indices between surfactant-free DNA films with thicknesses of 18 nm made by spin coating and 194 nm made by drop casting in the visible spectral range. (**b**) Refractive index as a function of the thickness of surfactant-free DNA films at λ = 632.8 nm. Error bars in (**a**) and (**b**) are the greater of standard errors in the ellipsometric fit and standard errors in the mean (SEM) of the measurements.

Before doping riboflavin, we investigated optical properties of pristine DNA films as a reference. DNA aqueous solution was spin coated on surface-treated silicon substrates to make thin solid film, as detailed in the ‘Methods’ section below. The optical dispersion of surfactant-free DNA thin films was measured by ellipsometry from 245 to 1690 nm using a Woollam M-2000 ellipsometer. The ellipsometric angles ψ and Δ were fit to an isotropic Kramers-Kronig consistent oscillator model^30^. From these fittings, we calculated the refractive indices for DNA thin solid films in the spectral range from 400 to 700 nm, as shown in Fig. 3(a). The thinnest film, prepared by spin coating, had a thickness of 18 ± 2 nm, and the thickest film, prepared by drop casting, had a thickness of 194 ± 5 nm. These samples were made from the same raw material and thin films were dried in identical conditions under vacuum yet we observed a significant difference in the refractive index of Δn > 0.045 over the entire visible spectral range. The thickness dependence of the refractive index was further investigated in the thickness range from 18 to 194 nm at the visible light wavelength λ = 632.8 nm. The relation between the film thickness and their refractive indices is shown in Fig. 3(b). In the figure, we observed that the refractive index of surfactant-free DNA solid film decreased with increasing film thickness up to a thickness of 100 nm. Beyond this thickness, the refractive index remained constant within the experimental errors up to the film thicknesses of 198 nm. In the film thickness range up to ~100 nm, the refractive index of DNA decreased at roughly −7.7 ± 1.6 × 10^−4^ nm^−1^, while beyond this limit the refractive index was observed to maintain around 1.5390 ± 0.0016. This is the first experimental report on the dependence of refractive index on film thickness for surfactant-free DNA films made from aqueous solutions, to the best knowledge of the authors.

Our observation for surfactant-free DNA films contrasts sharply with prior DNA-CTMA thin films^31^, where the refractive index increased with film thickness and then saturated in the bulk. In the case of DNA-CTMA films, this saturation behaviour has also been observed, and has been attributed to the orientation of the DNA aromatic rings perpendicular to the substrate. For the DNA molecule, it is likely that substrate interactions are higher in the thinner films, resulting in greater film orientation and tighter packing, and thereby a thickness-dependent refractive index. Likewise, the bonding of CTMA surfactants to the DNA phosphate backbone is known to reduce the optical density of the resultant thin film^14^ and may affect substrate interactions^32^. It should be noted that this thickness scaling applies for films fabricated to a given thickness, and that in biological environments, films will be subject to both degradation and hydration which will decrease both the film thickness and the refractive index^16,20^. The water content or OH radical bonding within DNA films made from aqueous solutions may also play a certain role in this surface interaction, and this role is being investigated by the authors.

### Optical absorptions due to riboflavin in surfactant-free DNA films

Doping of DNA-CTMA with conventional optical dyes such as Nile Blue and Rhodamine 6 G has been reported^31,33^, but neither of these prior papers^31,33^ reported in detail how the dopants vary the optical dispersion. These dyes are hazardous materials seriously affecting human organs upon direct exposure^34,35^. In contrast, vitamin B2-riboflavin is known to be highly biocompatible. In this study, we fully investigated the unique role of biocompatible riboflavin in optical dispersion control for surfactant-free DNA thin solid films.

**Figure 4:**
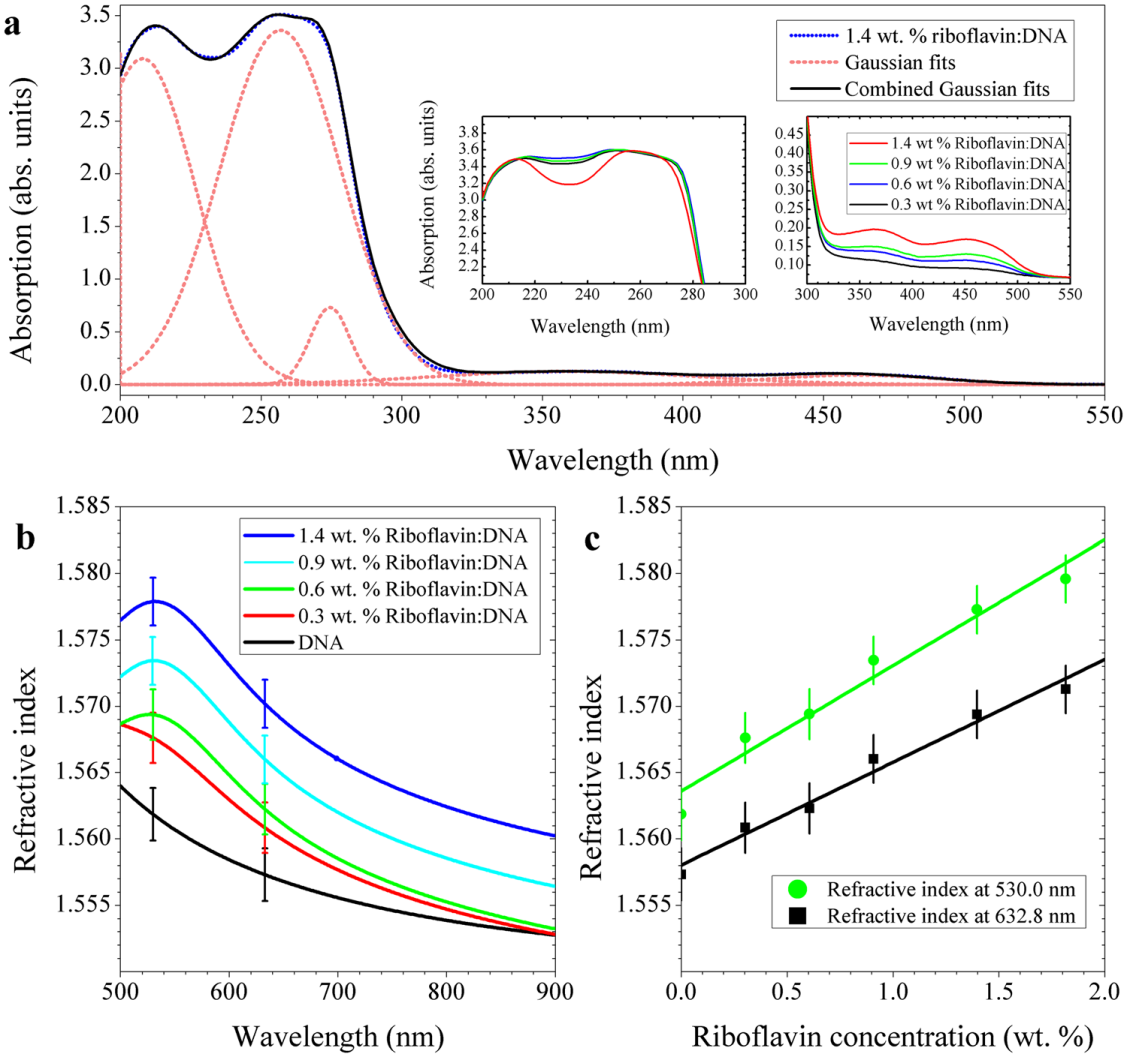
(**a**) UV-visible absorption spectra of riboflavin doped surfactant-free DNA thin solid films. The left and right insets are the absorption spectra in the UV and visible range for various riboflavin concentrations. (**b**) The refractive index of riboflavin doped surfactant-free DNA thin films for various riboflavin concentrations. (**c**) The refractive indices versus riboflavin concentration at λ = 632.8 and 530 nm. Error bars in (**c**) and (**d**) are the greater of standard errors in the ellipsometric fit and standard errors in the mean (SEM) of a number of measurements.

Accurate identification of optical oscillators in riboflavin doped DNA film is needed in order to establish an appropriate ellipsometric model. This allows us to adequately interpret the role of riboflavin in the optical dispersion control of surfactant-free DNA films. The films were made from aqueous solutions with DNA of 0.5 wt. % and riboflavin of 20, 40, 65, 100 μM, and we estimated the corresponding riboflavin concentration in the DNA film to be 0.3, 0.6, 0.9, 1.4 wt. %, respectively. The film thickness was measured to be ~200 nm. UV-visible absorption spectra were measured and the results are shown in Fig. 4(a). Absorption peaks were identified in the UV-VIS spectrum of the riboflavin-doped DNA solid film using Gaussian fitting, and are notated with dashed curves in Fig. 4(a). Peaks at λ = 207, 257, and 275 ± 5 nm correspond to characteristic oscillators of DNA^22,36^, and their spectral positions were almost independent of riboflavin doping, as seen in the left inset of Fig. 4(a). Riboflavin deposited on quartz substrates was measured to have absorption peaks in the UV-visible range, at λ = 349, and 451 nm (Supplementary Fig. 1). In Fig. 4(a), the peaks at λ = 358 and 461 ± 5 nm correspond to the absorption of riboflavin, and their strength showed a linear correlation with the riboflavin concentrations, as in the right inset of Fig. 4(a). It is noted that spectral locations of the riboflavin absorption peaks at λ = 349 and 451 nm in the solid riboflavin shifted to λ = 358 and 461 nm in DNA thin solid film. This strongly indicates that structural changes in riboflavin molecules and the subsequent electronic transitions occurred as the riboflavin molecules were immobilized into DNA films. Detailed structural binding characteristics of riboflavin are being further investigated by the authors and will be reported in a forthcoming paper.

### Refractive index control in surfactant-free DNA thin solid film by riboflavin doping

Ellipsometric models for riboflavin doped surfactant-free DNA films were modified by adding the two absorption peaks at 358 and 461 ± 5 nm for riboflavin as in Fig. 4(a), to calculate their refractive indices. All films had thicknesses of 45 ± 2 nm and the riboflavin concentration was varied in a range of 0.3, 0.6, 0.9 and 1.4 wt. %. In the visible and near IR spectrum, ellipsometric measurements were fit using a bootstrapping procedure^14^, as described in the following ‘Methods’ section. The resulting Kramers-Kronig consistent refractive indices are plotted for a series of riboflavin-doped DNA films in Fig. 4(b). Consistent increase in refractive index was observed as the riboflavin concentration increases. For the estimated riboflavin concentration of 1.4 wt. %, the index difference increased by Δn > 0.015 in reference to the pure DNA film. The refractive index of the DNA film was plotted as a function of the riboflavin concentration C_r_ in Fig. 4(c). Here we measured the refractive index at two visible wavelengths at λ = 632.8, the He-Ne laser wavelength, and λ = 530 nm, the peak of riboflavin fluorescence. The data were well-fitted in a linear regression and the observed refractive index increment slope was Δn/ΔC_r_ = 0.00775 ± 0.00007 (wt. %)^−1^ with respect to the riboflavin concentration at λ = 632.8 nm, and Δn/ΔC_r_ = 0.00950 ± 0.00006 (wt. %)^−1^ at λ = 530 nm. This refractive index increase is mandated by the Kramers-Kronig relations due to addition of riboflavin absorption peak**s** around λ = 358 and 461 nm, consistent with Fig. 4(a).

In conventional silica single mode optical waveguides, the refractive index difference between the core and cladding is Δn ~ 0.005 measured at λ = 632.8 nm (ref.^23^), which could be readily achievable in our DNA films by doping riboflavin less than 1.0 wt. %. See Fig. 4(c). It is also noted that the refractive index increases more rapidly in the shorter wavelength than infrared, which is due to the spectral proximity to the absorption peaks of riboflavin. Considering the relatively low loss beyond λ = 600 nm, as shown in Fig. 4(a), riboflavin-doped DNA core/DNA cladding waveguide structures can have a high potential in low-loss passive device applications in the red and near-IR spectral range. In the blue-green spectral range, meanwhile, the radiative transitions of riboflavin occur and we could expect an optical gain similar to recent reports in liquid solutions^24,25^ so all-DNA active solid state devices could be further pursued.

Nile Blue doping of DNA-CTMA thin films^31^ has shown a comparable refractive index variation within a maximum Δn of ~0.01. However, refractive index control in surfactant-free DNA film by riboflavin-doping can provide significantly more practical usages in biocompatible and eco-friendly applications.

### Thermo-optic coefficients of DNA and riboflavin-DNA thin solid films

**Figure 5:**
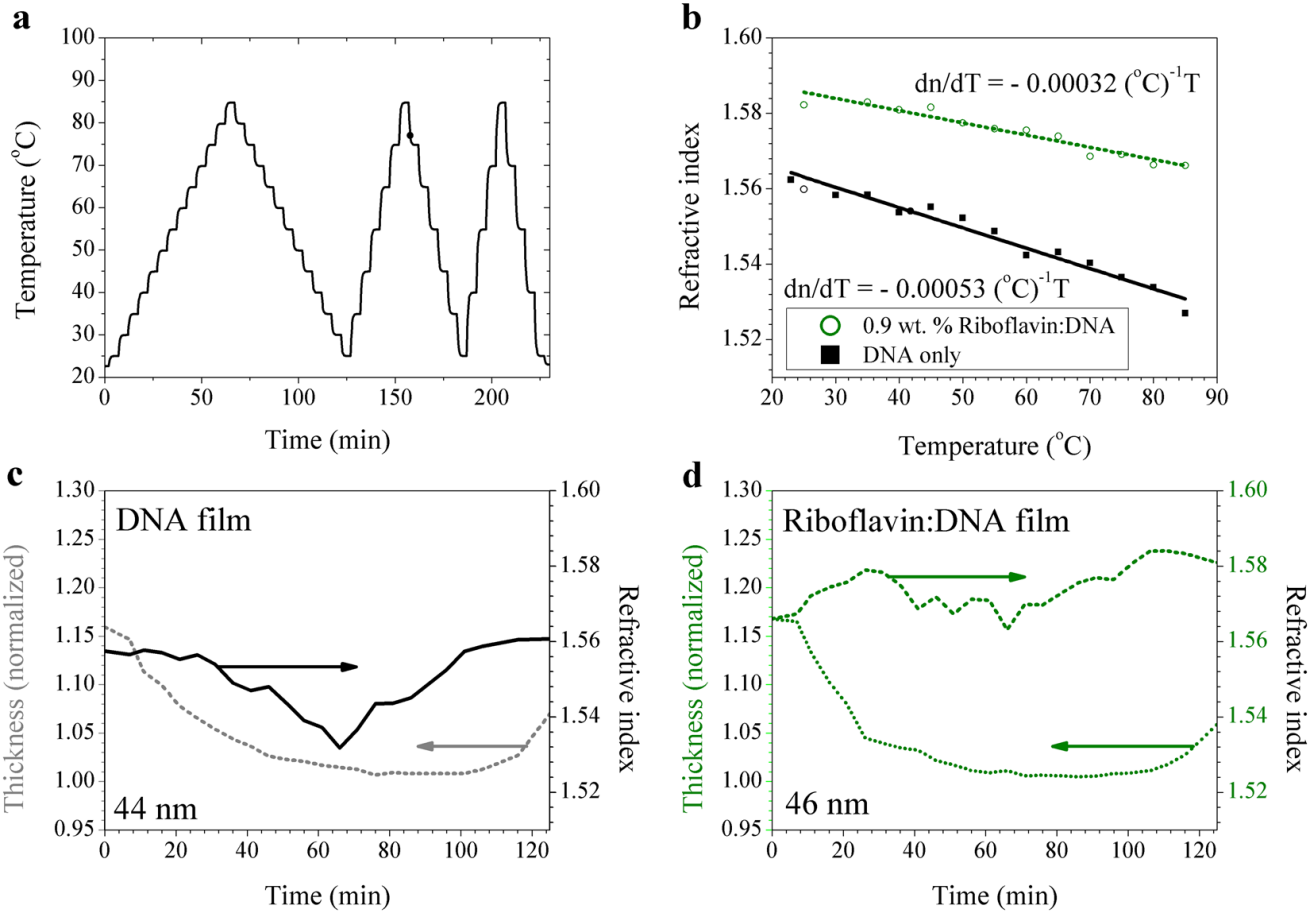
(**a**) Thermal cycles used in thermo-optic coefficient measurements. (**b**) Temperature dependences of the refractive index of DNA and riboflavin doped DNA thin film. The slopes of the linear fits represent thermo-optic coefficients, dn/dT. (**c**) Change in refractive index and relative thickness during the first heating-cooling cycle for 44 nm thick DNA film and (**d**) 46 nm thick 0.9 wt. % riboflavin-doped DNA film.

Thermo-optic response of both pure surfactant-free DNA and riboflavin-doped DNA films were investigated under the thermal profile shown in Fig. 5(a). Their film thicknesses were 44 nm and 46 nm, respectively. The concentration of riboflavin was estimated to be 0.9 wt. %. After the initial heating cycle, the refractive index change was observed to be linear, as shown in Fig. 5(b), and both films showed negative thermo-optic coefficients, dn/dT = −5.3 × 10^−4^ °C^−1^ for pure DNA and −3.2 × 10^−4^ °C^−1^ for riboflavin doped-DNA films. The absolute magnitude of the thermo-optic coefficient, |dn/dT|, in these surfactant-free DNA films was significantly larger than that of DNA-CTMA^37^, which has recently been measured to be dn/dT = −3.57 × 10^−4^ °C^−1^. Thermo-optic characteristics of DNA-CTMA film has been applied to a temperature sensor by depositing it over an optical fibre interferometer^37^, and the much larger thermo-optic coefficients of surfactant-free DNA and riboflavin doped DNA could further open thermo-optic device applications. We experimentally confirmed that by doping riboflavin into surfactant-free DNA films, we could achieve systematic control of both the refractive index and thermo-optic coefficient, which could provide a new high potential for all-DNA photonic devices and sensors.

Under thermal treatment at temperatures below the melting point of DNA, DNA-based biopolymers undergo changes in density and refractive index as a result of dehydration and polymeric unwinding^38^. During the first thermal cycle shown in Fig. 5(a), we measured variations in the thickness and the refractive index of DNA films and the results are summarized in Fig. 5(c,d). In both films, decrease of film thickness during the first temperature cycle is observed, which is consistent with prior DNA-CTMA thin films^31,36^. In the dotted curve of Fig. 5(c), the minimum thickness was referenced as unity and film thickness underwent changes from 1.15 to 1.0 and then back to 1.06 in the pure DNA film. In the riboflavin doped DNA film, the film thickness showed similar changes from 1.16 to 1.0 and back to 1.06, as depicted in the dotted curve in Fig. 5(d). Refractive index followed a pattern similar to the film thickness variation, but the final refractive index was slightly higher than the initial value, as in the solid curves in Fig. 5(c,d). Unlike in thin films of DNA-CTMA^31^, a region of positive thermo-optic coefficient was not observed after the initial heating cycle. The thermal behaviour of doped films of DNA-riboflavin resembles that of Nile Blue doped DNA-CTMA films^31^. Considering both Nile Blue doped DNA-CTMA and riboflavin doped surfactant-free DNA, we found that doping of the DNA-based host polymer magnifies the refractive index increment under initial heating cycle and then decreases the magnitude of the negative thermo-optic coefficient thereafter. This common behaviour likely reflects the role of dopants in compact packing and DNA strand immobilization, resulting in a reduced change in optical properties under thermal variation.

## Discussion

**Figure 6:**
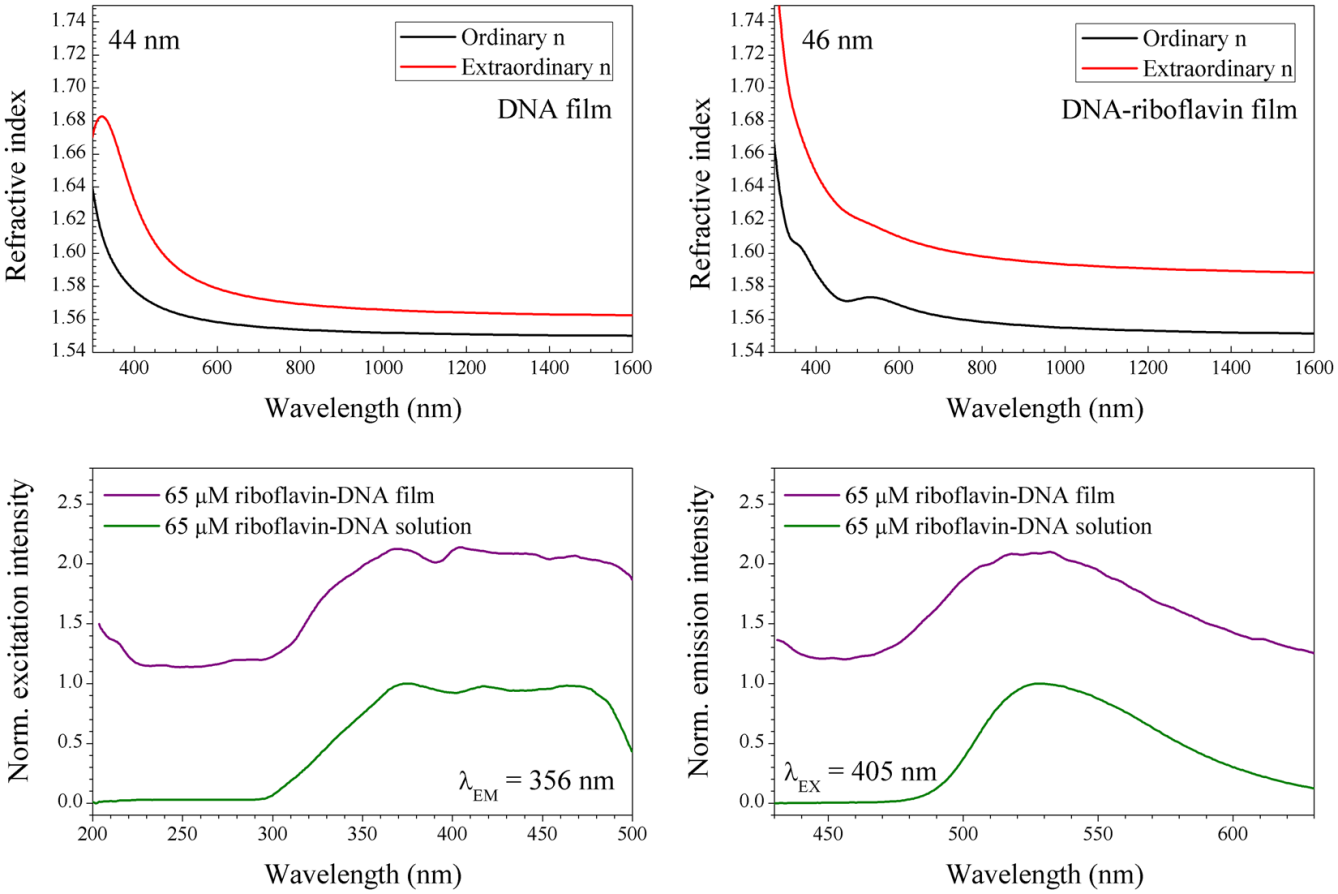
Refractive index as determined by spectroscopic ellipsometry using a uniaxial multiple-oscillator model (**a**) 44 nm-thick surfactant-free DNA film, (**b**) 46 nm-thick DNA thin film with 0.9 wt. % riboflavin doping. (**c**) Excitation and (**b**) emission spectra of riboflavin-doped DNA thin films (top) and aqueous solutions (bottom).

Isotropic fittings to the refractive index, as used for Figs 4 and 5, are generally accepted as a practical and useful tool for understanding overall dispersive trends in DNA thin films. However, anisotropy in the refractive index of DNA films has been recently quantified in thin films formed by slow evaporation^19–21,39^ and spin-coated DNA-surfactant thin films^14^. Therefore, we further investigated the anisotropic properties of our riboflavin doped surfactant-free DNA films using a uniaxial model. We followed the convention for the analysis of uniaxial materials on substrates, Δn_e-o_ = n_e_ − n_o_, where *n*_*e*_ and *n*_*o*_ are the refractive index in the extraordinary axis, and that in the ordinary axis, respectively^40^. The uniaxial ellipsometric fits to DNA and Riboflavin for films are summarized in Fig. 6(a,b), respectively. For un-doped DNA films, the amount of the optical anisotropy, Δn_e-o_, is in agreement with prior prism coupling measurements^20,21^, and is attributed to DNA strand alignment parallel to the substrate surface. For riboflavin-doped DNA films, we found a larger optical anisotropy, which suggests that the riboflavin assists in the horizontal orientation of DNA thin films. It is noteworthy that the oscillator amplitudes of the riboflavin absorption peaks near λ = 358 and 461 nm are also anisotropic, and occur preferentially in the ordinary component of the refractive index. See the black curve in Fig. 6(b). This suggests that riboflavin might be also oriented in the DNA host direction in thin solid films.

The fluorescence spectra of riboflavin in thin films and in solution were compared. Here we doped a 65 μM concentration of riboflavin (0.9 wt. % in thin films) into 0.5 wt. % concentration of DNA. Excitation and emission spectra in solid film and aqueous solution are summarized in Fig. 6(c,d), respectively. For the fluorescence at λ = 536 nm, excitation spectra were obtained and they showed similar spectral structures in both the film and the solution, as in Fig. 6(c). In thin film the excitation band was found to be slightly red-shifted, in a manner which mirrors that previously observed in films of riboflavin-doped silk due to binding with tyrosine and tryptophan^25^, and the bandwidth was broader than that in the solution. In the emission spectra in Fig. 6(d), fluorescence from thin film was also slightly red-shifted and significantly broadened, with a peak at λ = 532 ± 1 nm and its full width at half maximum (FWHM) = 92 ± 1 nm, compared with a peak at λ = 528.5 ± 1 nm and FWHM = 77 ± 1 nm in the solution. This spectral change indicates structural changes in the riboflavin molecule and subsequent electronic transitions within DNA solid film, consistent with changes in absorption spectra in Fig. 4(a). Fluorescence around 530 nm, combined with the observed increase in refractive index, offers a possibility of obtaining optical gain from a DNA-based riboflavin-doped optical waveguide device, which can provide high utility for all-DNA solid state photonic applications.

The present work has focused on the optical and thermo-optic properties of DNA and DNA-riboflavin films in their dry form. Previous research has shown that hydration of DNA films results in film swelling and decreases the refractive index^19,20^. Physical stability of the present thin films was observed up to relative humidities of 70%, and reductions in the refractive index were qualitatively observed with increased local humidity. Non-toxic films of DNA and DNA-riboflavin were soluble in water. DNA devices have been shown to be biodegradable^16^, which suggests applications in single-use biodegradable biomedical sensors or phototherapy devices, following further characterization of the optical properties of DNA films under humidity.

## Methods

### Material preparation

Aqueous solutions for the films were prepared by standard methods for spin-coating, similar to prior reports^18,19^. Dry filament of DNA-Na from salmon roe and milt (Ogata Research Laboratories, Ltd.) was purchased at 96% purity and at a molecular weight of ~10^6^ Da. Neat DNA was dissolved in 3.2 MΩ-cm deionized (DI) water by mechanical stirring in a sterile beaker to 0.5000 wt. % at pH 6.0 ± 0.2. Solution was mixed at 19~21 °C, and the solution was left at 4 °C overnight for preservation and removal of microscopic air bubbles. Riboflavin powder (Sigma-Aldrich) was dissolved in DI water by the above mixing process, and also stored at 4 °C. Riboflavin solutions at this low temperature were slowly mixed into the chilled DNA solution by a 60 rpm rotary mix for 3 minutes. DNA concentration in solution was 0.3500 ± 0.0001 wt. %, while the riboflavin concentration varied from 0 to 130 μM, as riboflavin concentrations were limited by precipitation and crystallization above 150 μM. As spin coating and DNA thin films are known to be very sensitive to viscosity, hydration, and process history^19,22,41^, special care was taken that precursor solution concentrations be accurate to one part in 10^4^ and that samples be subject to identical thermal, mixing, and humidity conditions.

### Thin solid film fabrication

We used a conventional spin coating procedure to deposit DNA thin films. Side-polished p-doped Si(100) wafers and sapphire substrates were used for ellipsometry and optical transmission measurements, respectively. They were washed for 10 minutes each in ultrasonic baths of acetone and isopropanol, and dried under flowing nitrogen. Substrates were then surface-activated by O_2_ plasma treatment, forming a 2.4 nm-thick silica layer on the Si wafer^42^, and stored in 1. kPa vacuum until spin coating. The above-mentioned precursor solutions were pipetted slowly onto prepared substrates, followed by spinning in a spin coater.

Films of pure DNA were prepared at spin speeds between 700 and 1000 rpm to obtain film thicknesses ranging from 20 to 70 nm. A drop casting method^43^ was also used to make thicker films with thicknesses up to 200 nm. For spin coating, DNA-riboflavin films were spun at a speed of 800 rpm, resulting in final thicknesses of 70 nm with variation of less than 3 nm across the film. Films were stored in vacuum at 1. kPa at room temperature for one day prior to optical measurements.

### UV-VIS spectrum analysis

Thin films of DNA and riboflavin-DNA were prepared on sapphire substrates and placed in the beam line with a 5° offset to minimize interference fringes. Scans accumulated three times on a JASCO V-650 spectrophotometer from 900 to 200 nm.

### Fluorescence spectrum measurement

Aliquots (3 ml) of the above solutions of DNA and riboflavin were prepared into 10 mm quartz cuvettes and scanned on a PerkinElmer LS-55 Luminescence spectrometer at 18 °C.

### Prism minimum deviation angle measurement

A hollow quartz prism with an apex angle of 30.28561° was filled with the solution to be measured. The angle of minimum deviation was measured with a digital goniometer (Moeller-Wedel Optical) using an HgCd vapour discharge lamp, from which the refractive index dispersion was calculated^28,44–46^. Solution temperature was recorded for every five angles measured, and ranged from 22.9 to 23.2 °C. Errors due to thermal variation were estimated to be ±0.0001.

### VASE ellipsometry

Ellipsometric angles were measured for fabricated thin films on two different ellipsometers: a Woollam α-SE spectroscopic ellipsometer for thermo-optic characterization, and on a Woollam M-2000U-NIR variable-angle spectroscopic ellipsometer (VASE). VASE measurements were taken at incident angles of 65.000, 70.000, and 75.000°, in the spectral range from 245 to 1690 nm (0.74 to 5.1 eV). VASE was measured with a collection time of 6 seconds at each incident angle at 23 ± 1 °C with a relative humidity of 38 to 41%.

A bootstrapping or iterative approach^14^ was used to confidently fit VASE spectroscopic ellipsometry measurements despite a large number of free oscillators. First, a Cauchy model was used in the transparent visible to IR regions to determine the likely film thickness and initial refractive index dispersion. An oscillator model using just the excitation bands of DNA thin film was then used to bootstrap this Cauchy model to an approximate fit to the refractive index, and to determine the likely size of the UV tail. For films of DNA, Gaussian oscillators were located at 6.50 eV, 4.74 eV, and 4.47 eV, and with a UV pole at 13.8 eV, matching energies previously observed^36^. For films of riboflavin-DNA, additional oscillators were added at 350 and 450 nm. All riboflavin peaks were initially fit across the set of all riboflavin samples to determine each oscillator’s energy band width, and then relaxed for each independent measurement. Finally, samples were fit individually with mutually coupled oscillator bandwidths. All fits were performed using a Levenberg-Marquardt algorithm and including depolarization data. This model resulted in confident fits to the observed spectrum of riboflavin-DNA, despite a lack of data for peaks in the far UV, all with mean squared error (MSE) less than 4 (ref.^47^).

### Thermo-optic coefficient measurement

Thin films of DNA and DNA-Riboflavin in atmosphere were placed on a closed-loop PID-controlled and water-stabilized Peltier thermoelectric device, and a thermal program was applied as shown in Fig. 6(a). The ellipsometric angles ψ and Δ were measured on a Woollam α-SE Spectroscopic Ellipsometer from 380 to 900 nm at an incident angle of 69.913°, while temperature was cycled between 25 to 85 °C. Cooling was achieved by heat diffusion to the water reservoir and to the ambient air. Ellipsometric measurements were repeated three times at each temperature in each cycle, and were fit in weighted batches to a Kramers-Kronig consistent constrained oscillator model derived from the VASE measurements using a Levenberg-Marquardt algorithm. Measurements used 30 second integration times and were performed in a climate-controlled laboratory at 22 °C and 23.0% relative humidity.

## Conclusion

A new method to systematically control the optical dispersion and thermo-optic properties of surfactant-free DNA thin films was developed by doping them with vitamin B2, also known as riboflavin. We established surfactant-free DNA thin film deposition processes based on spin coating of aqueous solutions on silicon and sapphire substrates, which allowed precise control of the uniform film thickness. The refractive index of surfactant-free DNA thin films decreased with film thickness up to 100 nm with a slope of roughly −7.7 ± 1.6 × 10^−4^ nm^−1^, while beyond this limit the refractive index was observed to maintain around 1.5390 ± 0.0016. This behaviour was opposite to the previously observed behaviour of DNA-CTMA and was attributed to thickness-dependent substrate interactions in the surfactant-free DNA films. In aqueous solutions, riboflavin was found to not have strong short-range interactions with DNA, resulting in a negligible change in the refractive index of the solutions. In surfactant-free DNA thin solid films, riboflavin was immobilized in DNA polymeric structures and increased the refractive index in a very linear manner with Δn/ΔC_r_ = 0.00775 ± 0.00007 (wt. %)^−1^ at λ = 632.8 nm, which enabled systematic and repeatable control of DNA film for all-DNA optical waveguide applications. Temperature dependent refractive indices of surfactant-free DNA film and DNA-riboflavin thin films were measured in the temperature range from 25 to 85 °C, and we obtained linear thermo-optic coefficients of dn/dT = −5.3 × 10^−4^ °C^−1^ for DNA and dn/dT = −3.2 × 10^−4^ °C^−1^ for 0.9 wt. % riboflavin-doped DNA films. In riboflavin-doped DNA films, we found a larger optical anisotropy, Δn_e-o_, than in the un-doped DNA film, which suggests that the riboflavin assists in the horizontal orientation of DNA thin films. We observed a red-shift and broader FWHM in the emission spectra of riboflavin in DNA thin film in comparison to riboflavin-DNA in aqueous solution. By doping riboflavin in surfactant-free DNA thin film, we were able to increase the refractive index and decrease the magnitude of thermo-optic coefficient, while maintaining its emission properties, thereby opening the possibility to fully biodegradable and biocompatible all-DNA optical waveguide devices and sensors.

## Electronic supplementary material

Supplementary Information
