# Supplementary material for: Optical dispersion control in surfactant-free DNA thin films by vitamin B2 doping
Source: Sci Rep. 2018 Jun 19;8:9358. doi: 10.1038/s41598-018-27166-x (PMC6008429; doi:10.1038/s41598-018-27166-x)
Supplement: Supplementary file 1 — Supplementary Information [file 41598_2018_27166_MOESM1_ESM.doc]

**SUPPLEMENTARY INFORMATION**

Optical dispersion control in surfactant-free DNA thin films by vitamin B_2_ doping

Bjorn Paulson^1^, Inchul Shin^1,2^, Hayoung Jeong^1^, Byungjoo Kong^1^, Reza Khazaeinezhad^3,4^, Sreekantha Reddy Dugasani^5^, Woohyun Jung^1^, Boram Joo^1^, Hoi-Youn Lee^6^, Sungha Park^5^, Kyunghwan Oh^1,*^

^1^Photonic Device Physics Laboratory, Institute of Physics and Applied Physics, Yonsei University, Seoul 120–749, South Korea

^2^Samsung Electronics, Hwasong, Gyeonggi-do 18448, South Korea

^3^Harvard Medical School, Boston, Massachusetts 02115, USA

^4^Wellman Center for Photomedicine, Massachusetts General Hospital, Boston, Massachusetts 02114, USA

^5^Sungkyunkwan Advanced Institute of Nanotechnology (SAINT) and Department of Physics, Sungkyunkwan University, Suwon 440-746, South Korea

^6^Space Optics Research Center, Korea Research Institute of Standards and Science, Daejeon 34113, South Korea

*koh@yonsei.ac.kr

*Corresponding author

Kyunghwan Oh

Fax: +82-2-365-7657; Tel: +82-2-2123-5608; E-mail: [koh@yonsei.ac.kr](mailto:koh@yonsei.ac.kr)

In order to characterize the potential interactions between DNA and riboflavin in thin films, we fabricated solid riboflavin for UV-VIS absorption spectrum measurement. Riboflavin precursor solutions were poured onto an untreated quartz substrate and left to dry for three days. The resultant deposition was highly non-uniform, with a coffee-stain type thickness distribution of riboflavin crystals. UV-VIS measurements were taken as in the rest of the manuscript, with the quartz background spectrum removed.

Supplementary Figure 1 displays the absorption spectrum of solid riboflavin in the UV-visible spectral range. Gaussian decomposition reveals significant absorption peaks at 223, 267, 349, and 451 ± 2 nm, with standard widths of 26, 50, 81, 118 ± 5 nm. This corresponds with redshift of about 10 nm in doping riboflavin into thin solid films of DNA. Significant scattering was observed and fit with a scalar baseline shift.

**
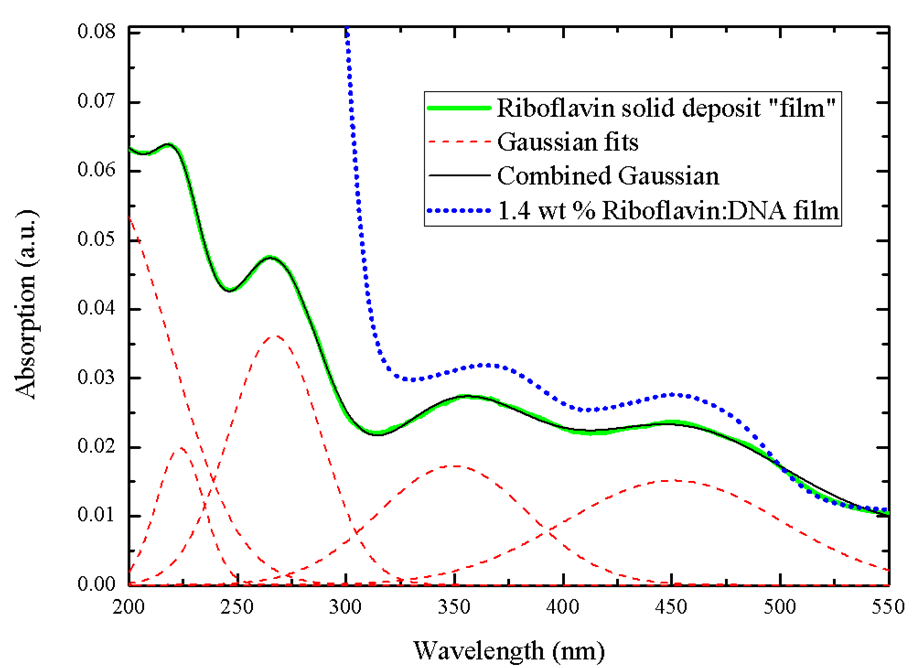
**

**Supplementary Figure 1.** UV-VIS Absorption spectrum of solid riboflavin deposited on quartz substrate. Riboflavin thickness was highly non-uniform, so 1.4 wt. % riboflavin-DNA film absorption was arbitrarily scaled for easy comparison.
